# Breast-Milk Substitutes: A New Old-Threat for Breastfeeding Policy in Developing Countries. A Case Study in a Traditionally High Breastfeeding Country

**DOI:** 10.1371/journal.pone.0030634

**Published:** 2012-02-09

**Authors:** Hubert Barennes, Gwenaelle Empis, Thao Duong Quang, Khouanheuan Sengkhamyong, Phonethepa Phasavath, Aina Harimanana, Emercia M. Sambany, Paulin N. Koffi

**Affiliations:** 1 Institut de la Francophonie pour la Médecine Tropicale, Vientiane, Lao People's Democratic Republic; 2 INSERM 897 VIH Afrique VIH, VIH, Cancer et Santé Globale dans les Pays à Ressources Limitées, Bordeaux, France; Indiana University, United States of America

## Abstract

**Background:**

Developing countries with traditionally breastfeeding are now experiencing the increasing pressure of formula milk marketing. This may endanger lives and undermine the efforts of national policies in achieving the objectives of the Millennium Development Goals. We examined the use of, and factors for use, of all available breast-milk substitutes (BMS) in a country with a traditionally high rate of breastfeeding.

**Methods:**

Randomised multi-stage sampling surveys in 90 villages in 12/17 provinces in Laos.

Participants: 1057 mothers with infants under 24 months of age.

Tools: 50-query questionnaire and a poster of 22 BMS (8 canned or powdered milk; 6 non-dairy; 6 formulas; 2 non-formulas).

Outcome measures included: prevalence of use and age of starting BMS in relation to socio-demographic characteristics and information sources, by univariate and multivariate analyses.

**Results:**

Of 1057 mothers: 72.5% currently breastfed; 25.4% gave BMS (10.6% infant formula); 19.6% gave BMS before 6 months of age (of them: 83% non-dairy or cereals; mean age: 2.9 months; 95% Confidence interval: 2.6–3.2). One formula and one non-formula product accounted for 85% of BMS. BMS were considered as milk by the majority of mothers. Thai TV was the main source of information on BMS for mothers. Lao Loum mothers (the main ethnic group) living in concrete houses with good sanitary conditions, were more likely than others to use BMS before 6 months (OR: 1.79, (1.15–2.78), p<0.009). Mothers who fed their infants colostrum at birth were less likely to use BMS before 6 months of age (OR: 0.63, (0.41–0.99), p = 0.04). Unemployed mothers living in rural areas were less likely to consider BMS better than breast milk.

**Conclusion:**

In Laos, mothers with the highest socio-economic status are showing a tendency to give up breastfeeding. Successful educational strategies and advocacy measures should be urgently developed to promote and sustain breastfeeding in developing countries.

## Introduction

Exclusive breastfeeding (EBF), an essential intervention to improve child survival [1], is the optimal way of feeding infants in the first 6 months of life [2]. According to UNICEF, considerable variation exists across regions ranging from 20% in West/Central Africa, 22% in Central and Eastern Europe and the Commonwealth of Independent States, 41% in Eastern/Southern Africa, and 43% in East Asia/Pacific where the highest rate of EBF is observed [3]. EBF rates increased from 34 to 41 percent between 1990 and 2004 for all developing regions for which there is data, except the East Asia/Pacific region.

For many years, it has been recognized that in unhygienic conditions breast-milk substitutes carry a higher risk of infection than breastfeeding and can be fatal for infants [4], [5]. The situation now is slightly worse, and not only due to poor hygiene.

Depriving infants and mothers of the benefits of breastfeeding is challenging all 8 Millennium Development Goals (MDGs) [4]. Breastfeeding is being endangered by the increased marketing of formula and non-formula milk [6]. Any food or drink given to an infant before six months of age is a breast-milk substitute (BMS), as optimal infant feeding is exclusively breastmilk until six months. After six months, anything used to replace that part of the infant's diet best fulfilled by breastmilk is a breastmilk substitute [7]. The advertising, though attractive, highly convincing and sometimes subtle, is thoroughly misleading. Funds dedicated to promoting breastfeeding are negligible compared to what is available for the advertising campaigns of companies manufacturing BMS [4].

The attractiveness of BMS is of concern in low income countries as these products become increasingly available [7]. Mothers feeding their children are attracted by the new products on display. They may not be able to tell the difference between appropriate and inappropriate products. This is an old but well-known problem that has not been resolved. The advertising and use of BMS has a strong negative impact on child survival.

Laos is a multi-ethnic and multilingual country with more than 45 languages. The official literacy rate (73%) does not reflect the disparity between the urban (89%) and rural areas (54%) [8]. Restricted diets during the post-partum period and inappropriate, or early, supplementary feeding of infants is common nationwide [9], [10]. Micronutrient deficiencies, beriberi (clinical vitamin B1 deficiency), and childhood bladder stones have been associated with low dietary diversity and the early feeding of glutinous rice to the newborn [11], [12].

Inadequate breastfeeding and weaning practices contribute to high rates of malnutrition and infant and child mortality [2], [9], [11], [12]. Inadequate nutritional practices and the food insecurity rates explain why the last decade has not shown any decrease in the high prevalence of stunting (41%) and wasting (15%) among children (0–5years) despite significant economic growth. [13], [14]. Stunting rates reach up to 65% in 12–23 month old children in the northern areas [15]. In Laos, over 95% mothers breastfeed their new-born but the rate of EBF is only 26.4% [16], [17].

We previously reported the misleading impact of a label on a coffee creamer which showed a bear suckling its cub. This resulted in 18% Lao mothers giving this creamer to their infants from the average age of 4.7 months in 2007 [18]. This attractive logo resulted in severe malnutrition and deaths, in both urban and rural areas [5], [19]. Our reports exposed the problem of repeated violations of the International Code on the Marketing of BMS in low income countries [7]. In Laos a wide range of BMS is available, promoted, displayed and sold next to infant formulas: coffee creamers; condensed and sterilized milk; together with infant formulas; follow-up formulas (such as Advance™); and cereals.

In this current survey we assess the prevalence of, and factors influencing, the use of BMS for Lao children less than 6 months of age. This survey is the first study to examine the use of BMS nationwide in a developing country.

## Methods

### Study procedure

**Figure 1:**
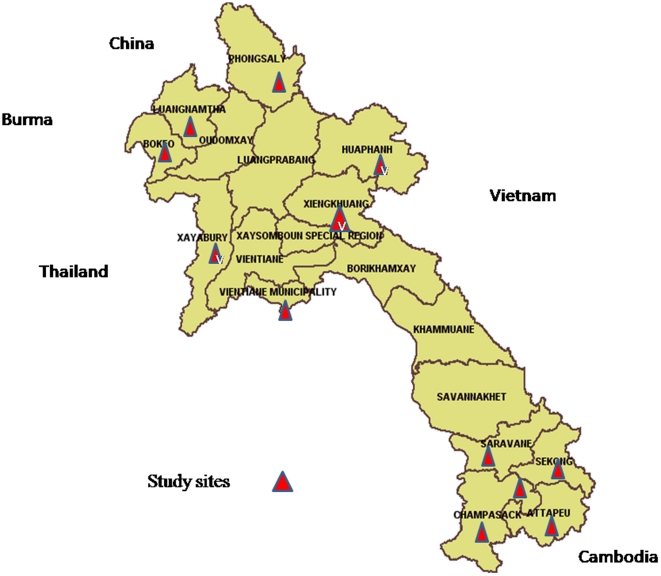
Map of study site.

Of the 16 provinces representing the geographical strata of Laos (North: from Phongsaly to Xayaboury, Luang Prabang and Xieng Khuang, Central (Vientiane and Borikhamxay), South (from Khammuan to Champassak) 12 were selected, based on the influence of bordering countries (China, Vietnam, and Thailand) (Figure 1). The number of provinces chosen was limited due to financial constraints. A multi-stage random sampling was applied in each province. The selection of districts and villages was undertaken prior to the investigation. In the first stage each district was allocated a number, and one district was randomly selected from each of the 12 provinces using a random numbers table. In the second stage random numbers generated by the table were used to select 8 villages from the list of all villages in each chosen district, and in the third stage, a systematic random sample was used to select 11 households from the list of households in each village. Mothers were included if they had a child less than 24 months of age. One mother per household was randomly chosen to be interviewed in Lao from all present household members more than 18 years. A translator was used if the mother was not fluent in the language.

Rural and urban areas were defined by the Lao government in the National Housing Census 2005 [8] which used five criteria to establish whether the village of residence was located in urban or rural areas. This was recorded in situ during the survey. The Census 2005 defined two categories for rural areas: those with access to roads and those without access to roads. In this survey the latter was limited to non-access within 4 to 6 hours on foot or by boat.

### Data collection

**Figure 2:**
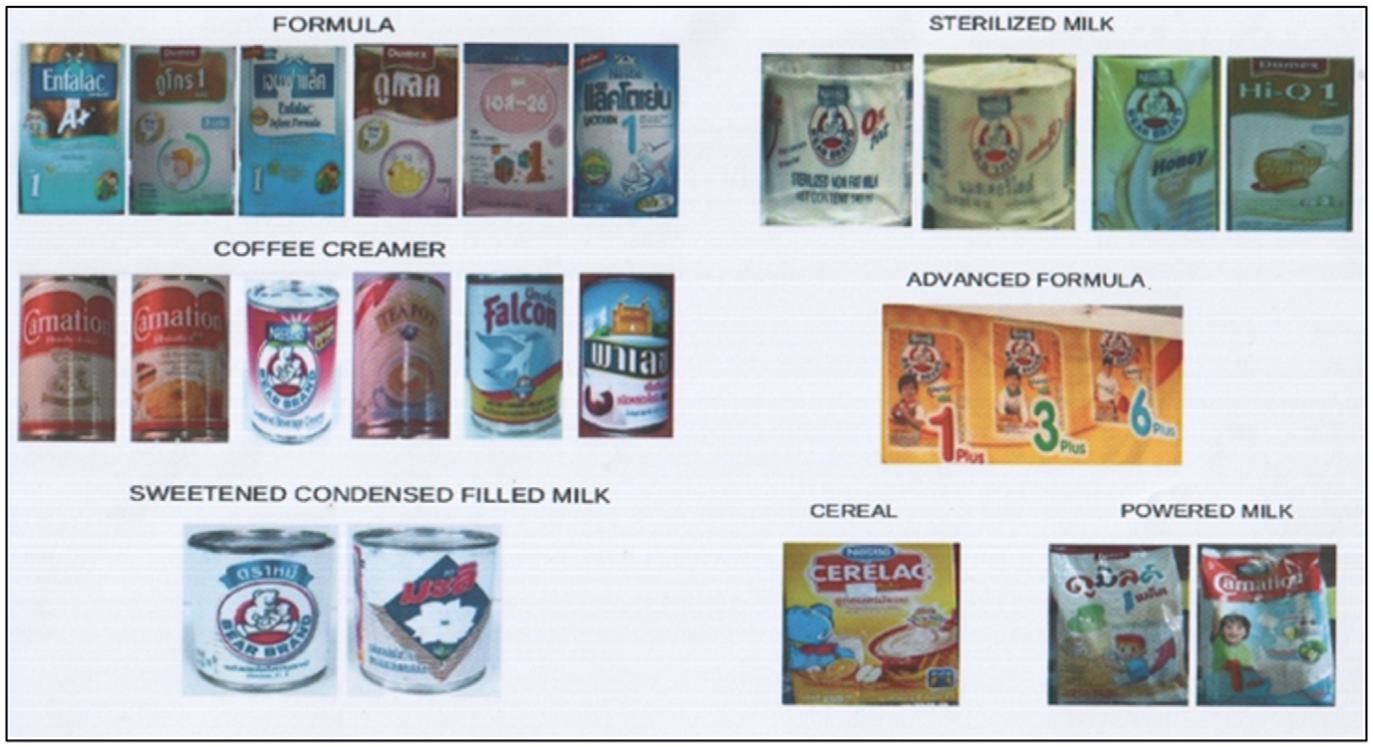
Pictures of the 22 available BMS in Laos.

A 50-query questionnaire examined the mothers' knowledge and usage of BMS available in Laos. Mothers were shown a poster with the pictures of 22 different BMS (Figure 2). The poster represented the majority of BMS available in the country according to systematic research conducted in the markets of Vientiane and the provinces prior to the investigation. Coffee creamers which are not BMS were also included in the survey since they are misused as BMS and displayed in shops alongside formula and BMS [18]. The following pictures were used: 6 coffee creamers, 6 infant formulas, 4 sterilised milk, 2 powdered milk, 2 condensed milk, 1 “follow-up formula” Advance™, and 1 cereal. The first 10 BMS were canned products, 2 were in tetrapaks and the rest were powder packed in cartons. The composition of the main non-formula BMS has been previously described [5], [18], [19]. Advance™ formula contains milk 58% (skimmed or full cream), carbohydrates 16–23%, (including honey, oligo-fructose and inulin), fat 13–22% (vegetable fat, butter oil). Information regarding the prices and usage duration of the products was collected from mothers who used them. The questionnaire was pre-tested with 20 mothers from the general population during several pre-testing investigations in Vientiane.

Data collection was conducted from 1 March to 4 April 2009. Investigators were students of the Institut de la Francophonie pour la Médecine Tropicale (IFMT, Vientiane, Laos) who were young doctors attending a 2-year master's course with special lectures on Epidemiology, Field Research and Public Health. They were fluent in some of the languages used in the different areas. They were trained in interview techniques prior to the field survey. Local facilitators fluent in Lao were used when needed.

### Main outcome and definitions

Main outcome measure was the prevalence of use, and age of starting BMS. Secondary outcomes were: knowledge of BMS and source of information on BMS; rate of BMS use before 6 months of age; reasons for use of BMS and its cost; rate of current breastfeeding; rate of breastfeeding within 24 hours; and EBF among infants 5–6 months.

Inappropriate breast-milk substitutes (IBMS) use was defined as the use of any of the BMS before the age of 6 months. EBF was estimated using the characteristics of breastfeeding the day before the interview. Breastfeeding (BF) was defined as EBF if the mother complied with 4 criteria: baby received BF the day before; baby did not receive any solid food; baby did not receive any other liquid or semi-liquid food.

### Sample size

Using Stata Version 8 (Stata Cooperation, College Station, TX), we calculated a necessary sample size of 1040 people based on an estimated 40% use of canned or fresh milk for infants in Lao reproductive health survey [20], a 5% precision with alpha = 0.05 and 90% power and 5% of anticipation for drop-outs or refusals.

### Analysis

Data was double entered with Epidata (www.epidata.dk, Odense, Denmark) and Stata, Version 8 (Stata Cooperation, College Station, TX). We performed analyses on people who recognised and had used at least one BMS. We used Student's test and analysis of variance (F test) for normally distributed continuous data and χ2 and Fisher's exact tests for categorical variables as appropriate. We considered P<0.05 as significant. The use of BMS before 6 months was evaluated in univariate analysis: child's gender; number and range of children; family size; ethnic group; residential area; mother's age; education; and occupation, religion, antenatal care, access to and source of information: TV, radio, monthly income and standard of living (type of house, transportation means, latrines, electricity, and safe water); feeding habits including colostrum feeding; and decision-makers in the family. Variables with p≤0.2 for BMS use before 6 months were then fitted into a multivariate logistic regression model with backward step-by-step analysis using odd ratios (Stata8) to evaluate them for IBMS use.

### Ethics statement

The survey was done in compliance with the Helsinki Declaration [21]. It was conducted with the agreement of the Ministry of Health and local and regional health authorities. Mothers gave their written/oral informed consent to participate in the survey according to their literacy ability. The survey was approved by the National Ethical Review Board of Laos.

## Results

Of 1057 mothers, 34.6% lived in urban areas, 42% belonged to ethnic minorities, 20% were illiterate and only 53% could speak Lao. Tables 1 and 2 provide the main characteristics of the mothers and their children. Of the 1057, 81.5% reported that they breastfed within one day of birth and 12.7% discarded the colostrum (Table 2), 21.3% gave food or drinks at birth: water 13.3%; masticated rice 3.2%; artificial milk 2.2%, others 2.5%. EBF among infants aged 5–6 months was 32% (95% CI: 23–42) with no observed difference between urban and rural areas (31.5% versus 30.6%, respectively; p = 0.6). Of the 1057, 72.6% currently breastfed their children and 25% gave at least one BMS. On average urban mothers gave BMS more frequently than rural mothers. Mothers fed their children (<24 months) the following BMS: cereals (10.8%); formula (10.6%); coffee creamer (3.9%); condensed milk (3.1%); follow-up formula Advance™ (3%); sterilized milk (1.2%); and powder milk (0.5%) (data not shown). Of the whole sample of 1057 mothers, 12% reported that they used it as a supplement and 7.5% as a substitute when the infant was less than 6 months.

**Table 1:** Main socio-economic characteristics of mothers in 12 provinces of Laos.

	Urban	Rural		Total
	366 (34.6%)	691 (65.4%)	*p*	1057 (100%)
Age (years)*	27.7 (27–28.4)	25.6 (25.1–26)	0.000	26.3 (26–27)
Ethnic Group				
- Lao Loum**	294 (80.3)	281 (40.7)	0.000	575 (54.4)
- Lao Theung**	29 (7.9)	174 (25.2)		203 (19.2)
- Lao Soung**	41 (11.2)	200 (28.9)		241 (22.8)
Illiterate	25 (6.8)	185 (26.8)	0.000	210 (19.9)
Can speak Lao	282 (77.1)	279 (40.4)	0.000	561 (53.1)
Husband illiterate	20 (5.5)	119 (17.2)	<0.000	139 (13.2)
Farmers£	112(30.6)	538(77.9)	<0.000	650(61.5)
Number of children*	2 (1.9–2.2)	2.5 (2.4–2.7)	<0.000	2.35 (2.3–2.6)
Ante-natal care££	326 (89.1)	467 (67.6)	0.000	793 (75.0)
Daily expenses for food£££	4.5 (4–5)	1.92 (1.8–2)	<0.000	2.8 (2.6–3)
Availability of tap water	277 (75.7)	382 (55.3)	0.000	659 (62.4)
Latrines	303 (82.8)	434 (64.8)	0.000	737 (69.7)
No electricity	17 (4.6)	254 (36.8)	<0.000	271 (25.6)
Have a TV	313 (90.2)	309(58.9)	0.000	622 (71.3)
Have a radio	78 (21.3)	122 (17.6)	0.1	200 (18.9)

*mean (95% confidence interval);

**This classification is used to roughly describe ethnic groups belonging to lowlands, midlands and highlands though people may have migrated since then from their original residency.

£ 834 (78.9%) reported an occupation: 73 (6.9%) civil servants, 60 (5.7%) shop keepers, 18 (1.7%) workers, 16 (1.5%) other occupations.

££ At list one visit.

£££ In US dollars: US = 8000 Lao kip.

**Table 2:** Characteristics of children surveyed in 12 provinces of Laos.

	Urban	Rural		Total
	366 (34.6%)	691 (65.4%)	*p*	1057 (100%)
Sex (Male)	188 (51.4)	364 (52.9)	0.7	552 (52.2)
Age (Months)*	11.5 (10.7–12.3)	11.7 (11.2–12.3)	0.3	11.64 (11.2–112)
- 0–6months	122 (33.3)	218 (31.5)	0.5	340 (32.1)
- 7–12 months	86 (23.4)	153 (22.1)	0.6	239 (22.6)
- 13–24 months	158 (43.3)	320 (46.3)	0.3	478 (45.2)
Breastfeed <24 hours£	311 (85)	550 (79.6)	0.03	861 (81.5)
Discarded colostrum£	46 (12.6)	88 (12.7)	0.3	134 (12.7)
EBF at 5–6 months**	13 (31.5)	19 (30.6)	0.6	32 (32)
Currently breastfed££	240 (65.6)	527 (76.3)	<0.000	767 (72.6)
- 0–6months	111 (91)	210 (96.3)	0.04	321 (94.4)
- 7–12 months	72 (83.7)	142 (92.8)	0.03	214 (89.5)
- 13–24months	57 (36.3)	175 (54.7)	0.000	232 (48.6)
Current use of BMS£££	132 (36.1)	137 (19.8)	<0.001	269 (25.4)
- 0–6months	31 (23.5)	34 (24.8)	0.02	65 (24.2)
- 7–12 months	38 (28.8)	43 (31.4)	0.01	81 (30.1)
- 13–24months	63 (47.7)	60 (43.8)	0.000	123 (45.7)

*mean (95% confidence interval);

**of 100 children aged 5–6 months 37 urban and 63 rural infants;

£ The following questions were asked: How many hours after the baby is born, did you start breastfeeding? Did you give the colostrum to your baby?

££ Do you still breastfeed your child?

£££ Yesterday, what food did you give your child?

Of 340 children who were less than 6 months during the survey 94.4% were currently breastfed: 15.9% received BMS as a supplement and 3.2% as a substitute.

Among the older 717 children (7 to 24 months) 14.2% were currently receiving BMS as a supplement and 14.2% as a substitute. Details of IBMS given before the age of 6 months are shown in Table 3. Of 1057 mothers19.6% gave one BMS before the age of 6 months, and 2.4% before the age of one month (data not shown) (83% of the BMS were non-dairy or cereals, 42% formula, 7.7% follow-up formula (response with multiple answers). Coffee creamer and condensed milk were less popular than cereals or formula (Table 3). BMS feeding was given by bottle 43.5%, or spoon 56.5%.

**Table 3:** Knowledge of and feeding BMS to infants less than 6 months in Laos£.

	Urban n = 366 (34.6%)	Rural n = 691 (65.4%)	*p*	Total n = 1057	Knows at least one BMS£
Ever used one BMS before 6 months	92 (25.1)	115 (16.6)	0.000	207 (19.6)	
Age of starting at least one BMS (months)*	5.8 (4.3–5.9)	4.53 (3.9–5.2)	0.1	4.8 (4.3–5.3)	
Advance ™	5 (1.4)	11 (1.6)	0.00	16 (1.5)	244 (23.1)
Age of starting (months)	6.77 (4–9.7)	5.46 (2.2–8.7)	0.5	6 (3.9–8.1)	
Cereals	42 (11.5)	59 (8.5)	0.1	101 (9.6)	294 (27.8)
Age of starting (months)	4.26 (3.6–4.9)	3.83 (3.4–4.3)	0.3	4.01 (3.6–4.4)	
Formula	55 (15.0)	32 (4.6)	0.00	87 (8.2)	340 (32.2)
Age of starting (months)	2.54 (1.7–3.3)	2.39 (1.8–3.0)	0.8	2.13 (1.8–2.5)	
Coffee Creamer	20 (5.4)	26 (3.7)	0.2	46 (4.3)	620 (58.7)
Age of starting (months)	2 (1.3–2.6)	2 (1.5–2.4)	1.	2.0	
Condensed Milk	6 (1.6)	15 (2.2)	0.7	21 (2)	541 (51.2)
Age of starting (months)	4.12 (0.2–8.0)	5.41 (2.4–8.4)	0.59	2.64 (1.8–3.4)	
Sterilized Milk	-	1 (0.1)	-	1 (0.1)	236 (22.3)
Powder Milk	0	1 (0.1)		1 (0.1)	142 (13.4)

*Mean age and 95% confidence interval,

£ knows at least one BMS in the category.

The following questions were asked: Did you give this product to your baby? If yes, How old was your child when you started giving this product to her/him (month).

BMS were more often recognized in urban areas: 77.5% of the mothers recognized at least one of the BMS: 94.8% vs. 68.3% (p<0.000) in urban and rural areas, respectively. BMS were usually considered as milk by the majority of mothers: 93.9% versus 92.6%, p = 0.4; in urban and rural areas, respectively. Among the non-dairy canned coffee creamer this rate reached 98% for Bear brand™ and 93% for non-Bear brand™. 29.6% of the mothers considered coffee creamer a good product for their babies. Among tetrapaks, Cerelac™ was usually well recognised as a cereal by 52% and had the lowest rate of being mistaken for milk (45%). Formulas and non-Bear brand™ canned BMS were infrequently mistaken for cereal by 2 to 7% of respondents. Characteristics of the five most commonly used products are shown in Table 4.

**Table 4:** Top five BMS used before the age of 6 months for infants in Laos.

	Total	Cost*	Duration of use^£^	Cost per day*
	n = 207 (%)			
Cerelac™	101 (48.8)	3.2	19	0.2
Lactogen™ Formula	75 (36.2)	7.1	12.4	0.6
Bear brand™ coffee creamer	30 (14.5)	0.8	7	0.1
Advance™**	22 (10.6)	7	6.9	1
Dumex™ Formula	14 (6.8)	10.2	11.1	0.9

*One US$ = 8,000 kip. Mean prices reported by mothers, ^£^ How long does one can or box last or box.

**Main component: Skimmed milk powder (36,5%), full cream milk powder (21,9%,) vegetable fat mix (12,6%), sucrose 7,7%, Maltodextrin 6,7%, honey 5,5%, lactose 3,2%, oligo-fructose (2,6%), butter oil (1,3%), vitamins and minerals premix (0,9%), soya lecithin (0,3%), fish oil (0,1%).

One formula and one non-formula product (Lactogen™ and Cerelac™) accounted for 85% of the products used before 6 months of age (Table 4). Formula was more frequently used in urban than rural areas. The daily estimated cost of BMS ranged from USD 0.11 to USD 1.02. Advance™ formula was more expensive for daily use than formula. Mean BMS prices were considerably higher in rural than urban areas for all products (5–10% for coffee creamer, 60% for Advance™), except for Lactogen™ (0.90%) and Cerelac™ (0.70%) which were lower (data not shown). Though no Bear brand™ coffee creamer was found in the 90 villages surveyed, it was the most recognized product (55.9%).

Commercials on Thai TV (26%) and shopkeepers were the main source of information on BMS (Table 5); only 2% mothers reported getting this information from Lao TV. However, health staff (70.9%) and experiences by elders (8.9%) were the most trusted sources for information on nutrition compared to TV (7.1%), radio (3.6%) or other members of the family (1.3%).

**Table 5:** Main source of information on breast milk substitute (BMS).

	Urban	Rural		Total
	n = 366 (%)	n = 691 (%)	*p*	n = 1057 (%)
- Heard from£	180(49.2)	108(15.6)	0.000	288(27.2)
- TV Lao	10 (5,5)	10(9,2)	0.2	20(6,9)
- TV Thai	175 (97,2)	99(91,6)	0.001	274 (95,1)
- Shop keepers	18(4.9)	88(12.7)	0.000	106 (10)
- Friends	51(13.9)	53(7.7)	0.001	104(9.9)
- Health staff	13(3.5)	17(2.4)		30(2.8)
- Posters	10(2.7)	5(0.7)	0.009	15(1.4)
Trust at least in one source££	346 (94.5)	608 (88)	0.001	954 (90.2)
- Health Staff	280 (76.5)	469 (67.8)	0.003	749 (70.8)
- Older people	23 (6.2)	72 (10.4)	0.02	95 (8.9)
- TV Thai	29 (7.9)	22 (3.1)	0.001	51 (4.8)
- Radio	8 (2.1)	30 (4.3)	0.05	38 (3.6)
- Family members	11 (3.0)	17 (2.5)	0.6	28 (2.6)
- TV Lao	14 (3.8)	11 (1.6)	0.2	25 (2.3)
- Mother	5 (1.3)	10 (2.3)	0.9	15 (2.1)
- Husband	7 (1.9)	6 (0.8)	0.1	13 (1.2)

£ The following questions were asked: How did you hear about these products? (many answers possible), if the answer was TV: Which channels?

££ The following question was asked: Which of the above mentioned sources of information do you trust the most for your family?

Mothers used BMS because they believed the products were a good nutritive complement to breast milk (Table 6).

**Table 6:** Main reasons for giving BMS to infants before 6 months of age among users.

Reasons£	Urban	Rural		Mothers
	n = 89	n = 114	*p*	n = 203*
Advised by relatives and family	21 (23.6)	23 (20.1)	0.5	44 (21.6)
Safe for baby	15 (16.8)	22 (19.3)	0.6	37 (18.2)
Nutritional value**	20 (22.4)	17 (14.8)	0.1	37 (17.4)
Complementary feeding	17(19.1)	31 (27.6)	0.1	48 (23.8)
Work (too busy to breastfeed)	11 (57.8)	8 (7.1)	0.2	19 (9.3)
Cannot breastfeed	4 (4.4)	10 (8.7)	0.1	14 (6.9)***
Health staff advice	6 (6.7)	8 (7.0)	0.9	14 (6.9)
Easy to use	3 (3.3)	6 (5.2)	0.7	9 (4.4)
Infant loves it	4 (4.4)	4 (3.5)	0.7	8 (3.9)
Weaning	3 (3.3)	2 (1.7)	0.4	5 (2.4)
Advertising	3 (3.3)	2 (1.7)	0.4	5 (2.4)
Cheap	2 (2.25)	0	0.1	2 (1)

£ The following question was asked: Why do you feed your baby this product?

*Of 207 users before 6 months, 203 users ‘responses available.

**Rich with vitamins, similar to rice, good for children.

***1.4% of 1057 mothers reported that they could not breastfeed.

Mothers with no education, but having a radio were 4.5 and 2 times more likely to consider breast milk better than BMS. Unemployed mothers living in rural areas were less likely to consider BMS better than breast milk (multivariate analysis, data not shown). The multivariate analysis between selected independent variables and BMS used before 6 months is shown in Table 7. Lao Loum mothers, the main ethnic group, living in concrete houses with good sanitary conditions, were more likely to use BMS before 6 months. Also mothers who fed colostrum to their newborn were less likely to use BMS before 6 months of age.

**Table 7:** Factors associated with the use of BMS before 6 months of age in Laos (multivariate analysis).

Ever used one BMS before 6 Months	OR	95% CI	*p*
Lao Loum	1.8	1.2–2.8	0.01
Buddhist	1.8	1.1–2.9	0.01
House made of concrete	1.6	1.1–2.5	0.02
House with latrine	1.5	1–2.2	0.07
Mother breastfed colostrum	0.6	0.4–1	0.05

95% Confidence Interval.

## Discussion

Optimal breastfeeding of children under 2 years of age has the potential to help prevent 1.4 million deaths annually in children under 5 in the developing world [22]. The rate of exclusive breastfeeding in East Asia (the highest worldwide) over the last 15 years remains unchanged though the global trend is increasing [23]. This survey highlights some of the reasons that explain the situation and why it is important not to consider breast-milk substitutes as the same, old problem. Traditional breastfeeding countries and societies similar to Laos are facing new and unexpected challenges with the introduction of a wide range of BMS. In Laos the traditional habit of giving masticated glutinous rice on the first day of life is no longer the only important reason for low EBF rates [9].

This may still impede the objectives of the Millennium Development Goals. This survey provides baseline data on BMS use and suggests some clues on how to improve promotion of breastfeeding in countries similar to Laos.

In this survey we found some differences between values reported in the Laos Reproductive Health Survey (LHRS) and the National Maternal and Child Nutrition Survey (MICS survey) which could probably be explained by the higher proportion of urban mothers, and the restricted access to remote rural areas in this survey (urban: 34.6% versus 28% in MICS and 9.8% in LRHS) [17], [20]. The rate for children currently breastfed was very similar to the MICS survey and LRHS. However the survey is in agreement with some important characteristics of the Lao population reported in Census 2005 such as ethnicity (55% Lao Loum), rural residency (73%), and literacy rates (68 to 78% within this age group of women) [8]. This, together with the fact that only mothers with children less than 24 months of age were selected suggest less recall bias regarding breastfeeding habits. We chose not to present criteria such as the timely initiation of breastfeeding within an hour of life and used four criteria to evaluate the EBF the day before.

Among children less than 6 months mothers used BMS more often (15.9%) as a supplement than as a substitute (3.2%). However, after 6 months the use of BMS as a substitute increased to a similar rate (14.2%). Mixing BMS with breastfeeding can hamper and reduce the quality and duration of breastfeeding [24]. Providing a breast-fed child with supplements under the conditions which prevail in much of the developing world is potentially dangerous, whatever the source of the food. Low exclusive breastfeeding in low income countries such as Laos is a major concern. The official EBF rates did not change between 2000 and 2006 (23% and 26%, respectively) [23], [25]. In this survey, exclusive breastfeeding at 5–6 months was higher than previously reported [17]. However, even if Laos EBF appears to be greater than in the neighbouring countries of Vietnam and Thailand (5% and 17%, respectively), another survey in Vientiane Province in 2007 suggests a decreasing rate (19.4%) in areas close to the capital [16], [25]. This could forecast future declines in Laos, if no action is taken to face the increased use of BMS.

A quarter of the population were currently feeding their children with at least one BMS and 20% reported starting before 6 months (at least one non-formula: 83%). Of concern is the fact that some BMS were non-dairy products. The use of BMS may have hampered the EBF rate and benefits and shortened the well-established tradition of long durations of breastfeeding [24].

The 2009 survey which only interviewed mothers with children less than 2 years of age (versus 63.6% of mothers in 2007 survey) probably had less recall bias than the 2007 survey exploring the use of coffee creamers. The current survey, which includes more provinces (12 versus 5), more rural (65.4% versus 51%) and more illiterate people (20% versus 10%) is also probably more representative. It shows a decreasing trend in the use of coffee creamer for infants previously described (3.8% in 2009 versus 18% in 2007, p<0.001) which is a real improvement [18]. The distribution of the Bear brand™ coffee creamer was stopped in February 2008 [6], [26]–[28].

There seems to be no replacement by other coffee creamer or condensed milk brands in Laos. The other brands are labelled with pictures unrelated to mothers and children (such as tea pots, flowers, cakes etc). This supports our previous statement that the Bear brand™ was highly misleading to the population. We, and others, reported additional obvious violations of the International Code on the Marketing of Breast-milk Substitutes [5], [6], [18], [23], [29].

The Bear brand™ coffee creamer remains popular and the most recognized product by mothers. The licensed trade mark using the misleading Bear brand™ label is still widely available in Laos and Thailand and remains a cause for great concern [5], [6].

Financial reasons (daily food expenses per family: USD 2.00 versus USD 4.20 for rural and urban, respectively) and lower accessibility to BMS in rural areas explain the higher rate of BMS use in urban areas. An important reason is the higher exposure to misleading advertising from Thai TV that we found in urban and rural areas. Mothers repeatedly exposed to advertisements for infant formula expressed interest in buying them [16]. Promotion of infant formula in the media has a strong impact on breastfeeding practices [24], [30] and is associated with the decline of breastfeeding in Asia [28].

Lao mothers are highly influenced by their relatives in the decision making process for/against breastfeeding. Among relatives, involvement of the child's father can influence breastfeeding choices [16]. Though Lao mothers regarded health staff highly, they reported that they would always follow family advice since it was only the family that would help in the event of a serious problem (H. Barennes and K. Vongphayloth, unpublished data). Families and close relatives should be the focus of any campaign regarding breastfeeding promotion.

Mothers who fed colostrum to their babies (a recent achievement of the past 10 years) tended to use BMS less. Antenatal care was higher than 70%. This, and the high trust in health staff reported in the survey, confirms the role of antenatal care in Laos. More attention should be paid during antenatal care to allow mothers to make an informed choice on breastfeeding. Fathers should be invited to participate in antenatal care and educational programmes, thus encouraging them to play a more supportive role in breastfeeding. This should be encouraged in the community. Hospital staff have a positive influence on breast feeding practices but this intervention has to be reinforced in the community to ensure long term sustainability [31], [32].

The final multivariate analysis suggests that mothers with higher living standards were at higher risk of using BMS. Nearly all of them reported that they believed that BMS was real milk. They considered these products a good nutritive complement to breastfeeding. However, they had different feelings regarding the quality of the products. Cereals and formula which are attractively packaged, and highly priced, were the first choice and canned products the last. Advance™ follow-up formula, displayed in a similar manner to cereals and formula, was among the last choice (Table 4).

In developing countries substituting breastfeeding, to avoid, for example HIV transmission, is nullified by increases in non-infectious child mortality resulting in no net benefit for HIV-free survival [33]–[35]. Our survey showed a very low rate of mothers who stated they could not breastfeed (7% among the 207 BMS early users, only 1.4% of the total sample). The survey did not address the substituting of breast feeding by supplements to avoid HIV transmission. HIV prevalence is still very low and substituting breast feeding by supplements to avoid HIV transmission is not recommended policy in Laos. In fact practices may differ: contrary to Lao policy, in some hospitals infants of HIV positive mothers are given formula, free of charge and are told not to breast feed (L. Srour, personal communication). This survey limitation will have to be taken into account before generalising these findings and issues to other settings with higher prevalence of HIV.

The poor knowledge of mothers regarding BMS suggests that EBF could improve through nationwide information campaigns. Breastfeeding should be promoted through TV advertising and breastfeeding campaigns. Such intervention resulted in the improvement of breastfeeding practices in the Philippines [36]. In October 2009, a new exclusive breastfeeding campaign was launched in Laos. The current campaign features an unscrupulous formula marketer, dressed in western clothes, trying to take the baby off the mother's breast and offer it BMS [37].

Health authorities should focus on urban mothers but persist in maintaining appropriate messages towards the rural population as well, to prevent a decrease of breastfeeding, similar to that observed in the region. Important measures can be taken to enforce the International Code of Marketing of Breastmilk Substitutes, which Laos endorsed at the 1981 World Health Assembly. Legislation requires good enforcement and the support of UNICEF and international organizations to guarantee that child health workers and health professionals are protected from exposure to BMS companies. A programme to support breastfeeding could include the following components: monitoring violations of the Code; increasing advocacy and information of EBF among health staff; families with breastfeeding mothers; the general population; teenagers; and during all reproductive health services. Labelling of BMS should be improved and translated into the local language. The display and sale of non-formula products next to products intended for infant feeding should also be prohibited. It is the responsibility of manufacturers to respect the Code in the neighbouring countries [29].

### Conclusion

Inappropriate use of BMS for infants less than 6 months of age represents a threat to the preservation of the high breastfeeding rate in Laos and will hamper any improvement in EBF rates. Action should be taken to decrease the impact of misleading advertising, which influences mothers to switch from traditional breastfeeding to BMS. Enforcement of the International Code of Breastmilk Substitutes will protect and promote breastfeeding. Successful educational tools should be developed to promote, sustain and improve the quality of breastfeeding. Improved breastfeeding practices will save infant lives.
